# Identification of GSPT1 as prognostic biomarker and promoter of malignant colon cancer cell phenotypes via the GSK-3β/CyclinD1 pathway

**DOI:** 10.18632/aging.202796

**Published:** 2021-04-04

**Authors:** Xuan Long, Lijun Zhao, Guoqiang Li, Ziwei Wang, Zhigang Deng

**Affiliations:** 1 Department of General Surgery, Mianyang Central Hospital, Mianyang Central Hospital of Chongqing Medical University, Mianyang 621000, Sichuan Province, P.R. China; 2 Department of Gastroenterological Surgery, The First Affiliated Hospital of Chongqing Medical University, Chongqing Medical University, Yuzhong 400016, Chongqing, P.R. China

**Keywords:** colon cancer, bioinformatics, GSPT1, tumorigenesis, GSK-3β pathway

## Abstract

Colon cancer is the third most common malignant tumor and its mortality rate ranks fourth among all malignant tumor types. Bioinformatics analysis has shown that *GSPT1* is dysregulated in colon cancer and is associated with tumor progression. However, the underlying mechanism remains unclear. To address this research gap, we examined the impact of *GSPT1* on cell proliferation, apoptosis, migration, and invasion *in vitro* as well as tumor growth *in vivo* in colon cancer by using a Cell Counting Kit-8 assay, flow cytometry, transwell migration assay, transwell invasion assay, and tumor xenograft model-based analysis, respectively. *GSPT1* was significantly up-regulated in colon cancer tissues and cell lines. High GSPT1 expression was correlated with a larger tumor size. Depletion of GSPT1 suppressed cell proliferation, migration, and invasion-induced colon cancer cell apoptosis *in vitro* and restrained tumorigenicity *in vivo* in HCT116 colon cancer cells. Taken together, our findings suggest that the GSPT1/GSK pathway exerts tumor-promoting actions in colon cancer oncogenesis and progression. The GSPT1/GSK pathway may thus be an effective target for controlling colon cancer.

## INTRODUCTION

Colon cancer is characterized by malignant tumors in the digestive tract occurring at the junction of rectum and sigmoid colon [1–3]. Colon cancer is common and its incidence rate is highest in the 40–50 year old age group. Despite great advances in early diagnosis and combination therapy, colon cancer recurrence remains a challenging clinical issue [4–6]. Patients with metastatic colon cancer have a high incidence of recurrence within three years after initial treatment.

The incidence of colon cancer is mainly related to a high fat and low cellulose diet. Individuals with chronic inflammation of the colon have a higher incidence of colon cancer than the general population [7–9]. The incidence of colon cancer is five times higher in individuals with polyps than in those without polyps [10]. Genetic factors may also be involved in the pathogenesis of colon cancer. Genes involved in these pathological alterations should be considered as potential targets for the diagnosis or treatment of recurrent colon cancer [11–13].

In a previous study from our group, we analyzed colon cancer sequencing data in the cancer genome Altas (TCGA), mined the differentially expressed genes in normal colon tissues versus colon cancer tissues, and then analyzed the biological processes associated with the differentially expressed genes. One identified gene was *GSPT1* (G1 to S phase transition 1), which plays an important role in the biological process of colorectal cancer. As shown by the UCSC genomic browser, *GSPT1* is located in the long arm 13 region of human chromosome 16 (chr16 p13.13) and consists of 10 exons in the protein coding region. During transcription, *GSPT1* forms two different transcripts through variable splicing, which translate and encode 508 amino acids. *GSPT1* plays an important role in the genesis, progression, and prognosis of colon cancer. However, the biological function and molecular mechanism of GSPT1 in colon cancer remain unclear. In the present study, cell biology, pathology, and molecular biology technology are used to clarify the biological function of GSPT1 in colon cancer. Specifically, high-throughput Immunoprecipitation mass spectrometry (IP-MS) analysis is used to identify the target gene regulated by GSPT1 and the molecular signal pathway involved.

## RESULTS

### GSPT1 expression is up-regulated in colon cancer and cell line

**Figure 1:**
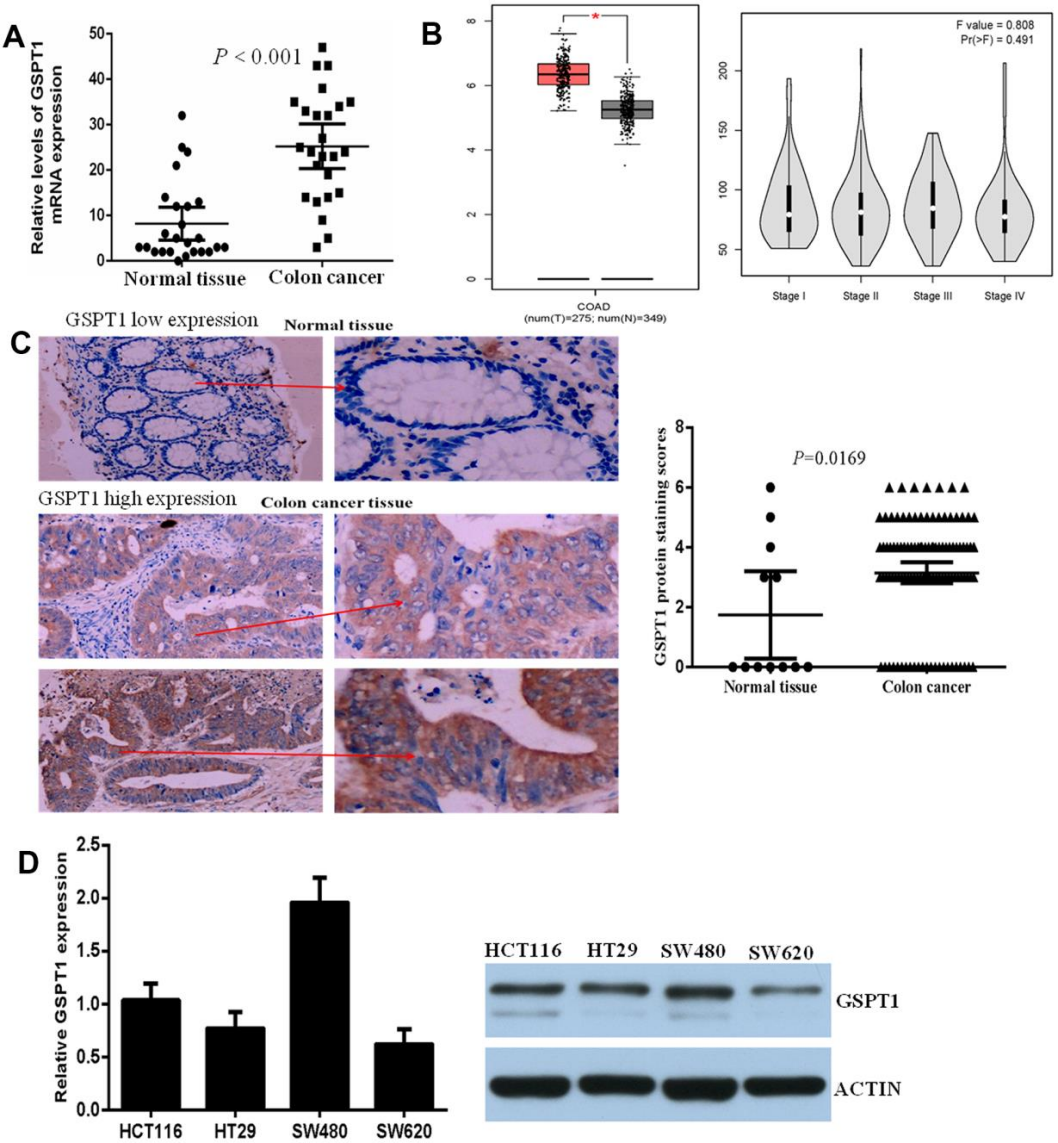
**GSPT1 was increased in colon cancer tissues and cell lines.** (**A**) Expression of GSPT1 in the colon cancer tissues and normal tissues were examined by RT-P assay. (**B**) The expression level of gspt1 in TCGA database. (**C**) Immunohistochemistry was used to detect the expression of GSPT1 in normal colon and colon cancer. (**D**) Expression of GSPT1 in the colon cancer cell lines were examined by RT-P and western blot assay.

The expression level of *GSPT1* mRNA was assessed by quantitative P in 25 cases of colon cancer and corresponding adjacent tissues. Statistical analysis showed that the expression of gspt1 in colon cancer tissue was significantly higher than that in normal colon tissue (*P*<0.001, Figure 1A). Based on colon cancer data in TCGA, we analyzed the expression level of gspt1 in colon cancer patients and controls. The results showed that the expression of *GSPT1* was significantly higher in colon cancer patients. Among colon cancer patients, the expression level of *GSPT1* gradually increased from stage I to II to III cancer, and decreased slightly in stage IV (Figure 1B). To examine the expression of GSPT1 in colon cancer tissue, tissue samples from 108 cases of colon cancer and 12 normal colons were collected. Compared with normal colon, the expression level of GSPT1 was significantly increased in colon cancer tissues. Figure 1C shows representative examples of immunohistochemically stained sections for low (up) and high (down) levels of nuclear GSPT1. The mRNA level of GSPT1 was also determined in four colon cancer cell lines. As shown in Figure 1D, the HCT116 and SW480 cell lines showed significantly higher levels of GSPT1 than the HT29 and SW620 cell lines. Given these results, all subsequent functional studies were performed using the HCT116 and SW480 cell lines.

### Association between GSPT1 expression and clinicopathological parameters in colon cancer patients

A total of 108 colon cancer patients aged 21 to 79 years were included in the analysis. Of these, there were 33 females (30.5%) and 75 males (69.5.9%). To analyze the clinical relevance of GSPT1, patients were divided into two groups based on their median GSPT1 value: high (≥median) or low (<median). Chi-square tests of patients’ clinicopathological characteristics showed that high expression of GSPT1 was significantly related to larger tumor size (*p*=0.008), but not to stage (*p*=0.806), lymph node status (*p*=0.801), or grade (*p*=0.922) (Table 1). In general, aberrant GSPT1 expression in colon cancer suggests that GSPT1 is strongly correlated with colon cancer tumorigenesis and progression.

**Table 1:** Correlation of GSPT1 protein expression with clinic pathological data (Fisher’sExactTest).

**Pathological** **variables**	**Sample**	**GSPT1 IHC staining(%)**		**Pvalue**
		**Negative**	**Positive**	
Normal tissues	12	7(83.3)	5(16.7)	0.010 (*)
Primary tumours	108	23(21.3)	85(78.7)	
Stage				0.806
I+II	70	14(20.0)	56(80.0)	
III+IV	38	9(23.7)	29(76.3.)
Lymph node status				0.801
pN0	74	15(20.3)	59(80.7)	
pN1+	34	8(23.5)	26(76.5)	
Grade				0.922
1	21	4(19.0)	17(81.0)	
2	65	14(21.5.)	51(78.5)	
3	12	3(25.0)	9(75.0)	
Missing	10			
Tumour size				0.008(**)
T1-3	62	19(30.6)	43(69.4)	
T4	46	4(8.69)	42(91.3)	

### Silencing GSPT1 inhibits cell proliferation and induces apoptosis *in vitro*

**Figure 2:**
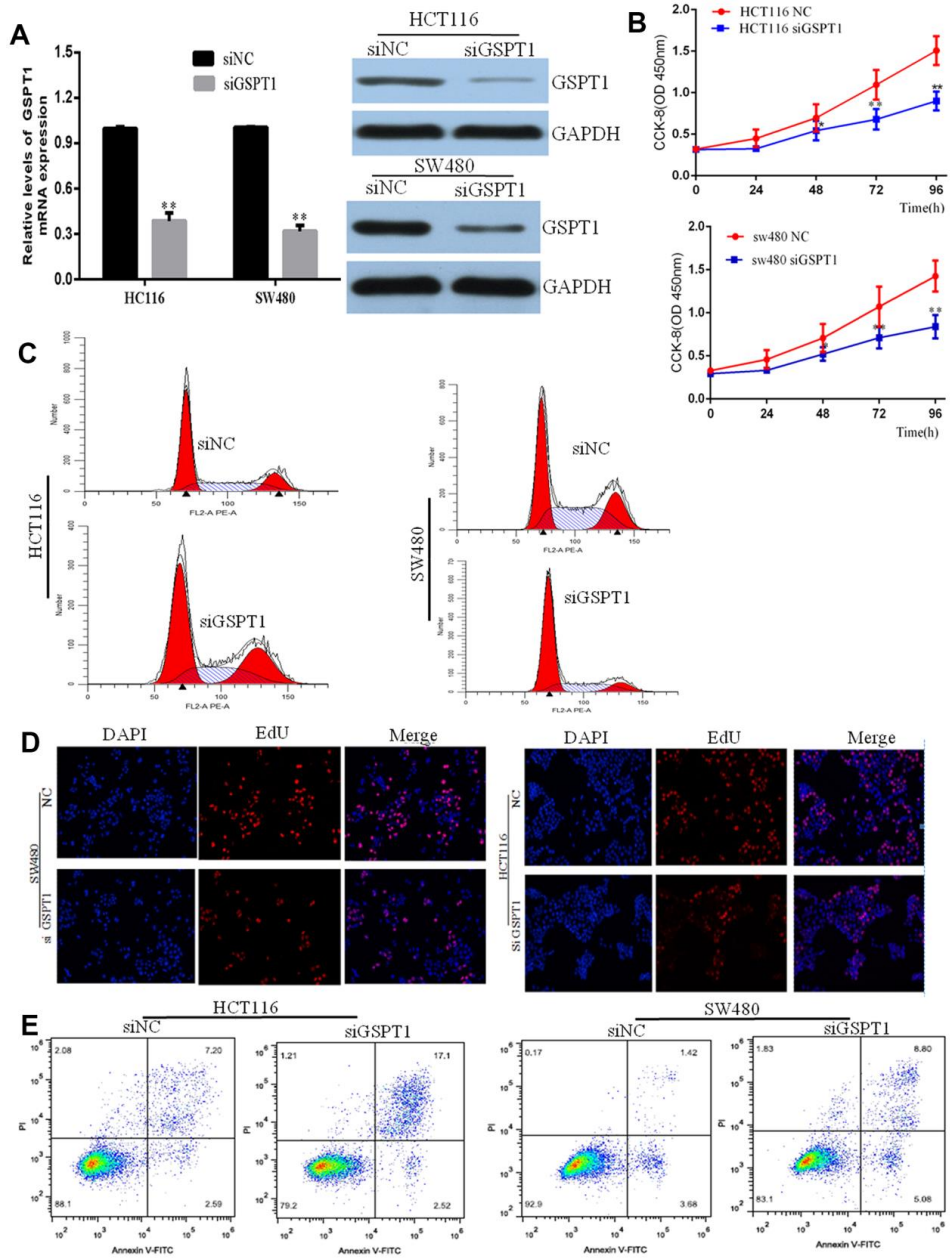
**Knockdown inhibits GC cell proliferation, colony formation, and stimulates apoptosis.** (**A**) Efficiency of siGSPT1 was evaluated by qRT-PCR and western blot. (**B**) HCT116 and SW480 cells were transfected with siGSPT1 or scramble control, and then assessed for cell growth rate at 48 and 72 h in CKK8 assay. (**C**) Effect of silencing GSPT1 on cell cycle of colon cancer cells HCT116 and SW480. (**D**) EdU assay to detect the effect of silencing GSPT1 on DNA synthesis of colon cancer cells HCT116 and SW480. (**E**) Apoptosis rate in the GSPT1 knockdown group was increased. ** *P* < 0.01.

To explore the molecular function of GSPT1, siRNA mediated *GSPT1* silencing was applied. Compared with control shNC (transfected with scramble control siRNA), *GSPT1* (transfected with *GSPT1* specific siRNA) reduced the GSPT1 level by over 50%, indicative of efficient gene silencing by *GSPT1* in HCT116 and SW480 cells (Figure 2A). We used the CCK-8 assay to evaluate cell proliferation and compared the results of the si-*GSPT1* and si-NC (nonsense siRNA) groups. After 24-, 48-, or 72-h incubation with siRNA, cell viability was assessed. No differences were found between the control and siNC groups at any of the three time points. Treatment with si-*GSPT1* significantly decreased *GSPT1* protein expression (Figure 2B). The flow cytometry results showed that, after *GSPT1* was silenced, the cell cycle of HCT116 and SW480 changed significantly: the proportion of G1 phase cells increased, the proportion of S phase cells decreased, and the proportion of G2/M phase cells did not change significantly (Figure 2C). Next, we used the EdU immunofluorescence assay to detect changes in DNA synthesis in HCT116 and SW480 cells after *GSPT1* silencing. The results showed that the proportion of cells in the S phase in the siGSPT1 experimental group was significantly lower than that in the siNC control group (Figure 2D). The effect of *GSPT1* expression on cell apoptosis in human colon cancer cells was also investigated. After treatment with nonsense siRNA (si-NC) or *GSPT1*-siRNA (si-*GSPT1*) for 24h, cell apoptosis was analyzed using flow cytometry. The result showed that the percentage of apoptotic cells in the control group (4.26±1.93%) and si-NC group (4.04±1.50%) were both significantly lower than that in the si-GSPT1 group (20.16±2.42%) (*P*<0.001) (Figure 2E), suggesting that down-regulation of GSPT1 induced colon cancer cell apoptosis.

### Over-expression of GSPT1 promotes the HCT-116 cell cycle and colony formation and inhibits cell apoptosis

**Figure 3:**
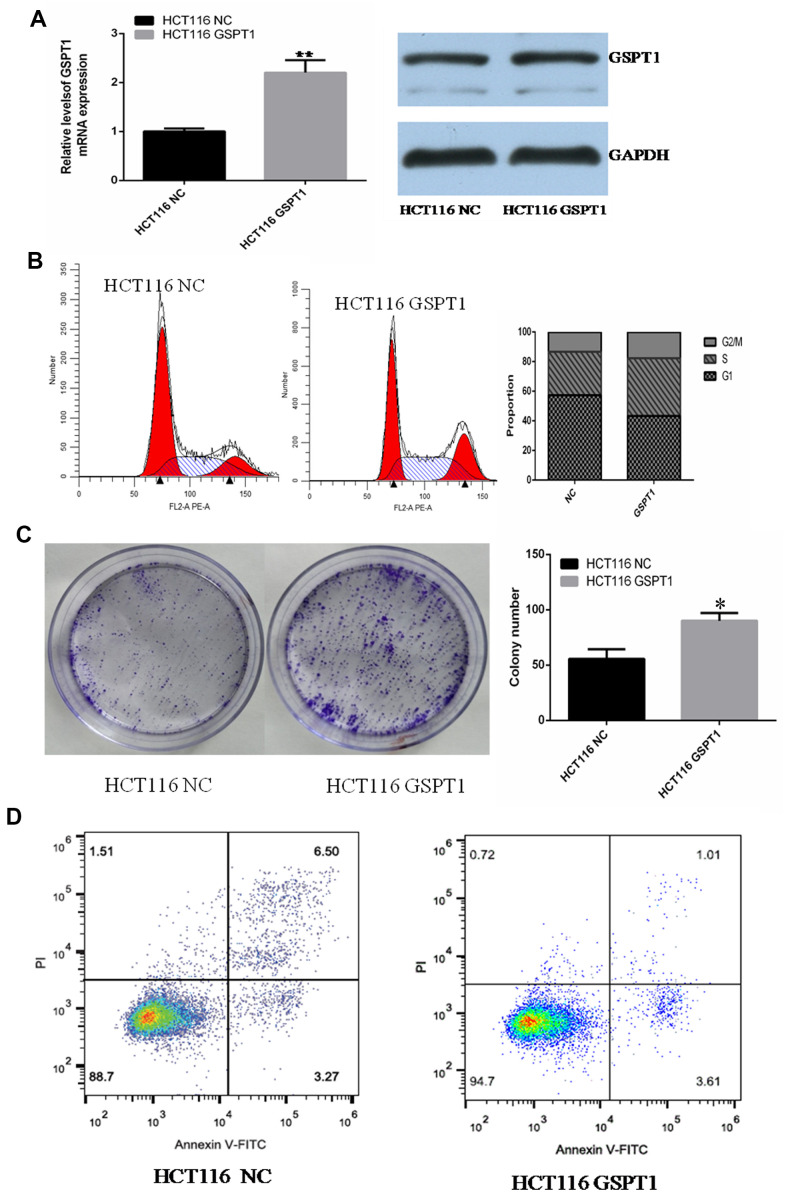
**Over-expression of GSPT1 promotes cell cycle progression and inhibits apoptosis in colon cancer cells.** (**A**) Quantitative PCR and WB were used to detect the over-expression efficiency of GSPT1 in colon cancer cell line HCT116. (**B**) Over-expression GSPT1 promotes cell cycle of colon cancer cell line HCT116. (**C**) Over-expression of GSPT1 promotes the cloning ability of colon cancer cell line HCT116. (**D**) Over-expression of GSPT1 inhibits the apoptosis of colon cancer cell line HCT116. * *P* < 0.05, ** *P* < 0.01.

To investigate the effect of *GSPT1* over-expression on the biological function of colon cancer HCT116 cells, the full-length sequence of *GSPT1* was cloned into a pcDNA3.1 plasmid and a *GSPT1* over-expression vector was constructed. After transfection into HCT116 cells, qRT-PCR and western blot confirmed that *GSPT1* was over-expressed in HCT116 cells as measured by mRNA and protein levels (Figure 3A). The flow cytometry results showed that, after *GSPT1* over-expression, the cell cycle of HCT116 changed significantly: the proportion of G1 phase cells decreased, the proportion of S phase cells increased, and the proportion of G2/M phase cells changed little. These results confirmed that over-expression of GSPT1 promotes G1 to S phase transition of colon cancer cells, and that *GSPT1* promotes progression of the colon cancer cell cycle (Figure 3B). Compared with the empty vector group, the number and size of cell clone formation of HCT116 cells were significantly increased after *GSPT1* over-expression (*P*<0.01), which confirmed that *GSPT1* promotes the proliferation of colon cancer (Figure 3C). After over-expression of *GSPT1*, apoptosis was detected by the AnnexinV/PI double staining method. The results showed that the apoptosis rates for the control group and over-expressed *GSPT1* group were 9.77% and 4.62%, respectively. These results confirmed that over-expression of *GSPT1* inhibited apoptosis in HCT116 cells (Figure 3D).

### Silencing of GSPT1 inhibits HCT-116 cell colony formation and invasion *in vitro* and *in vivo*

**Figure 4:**
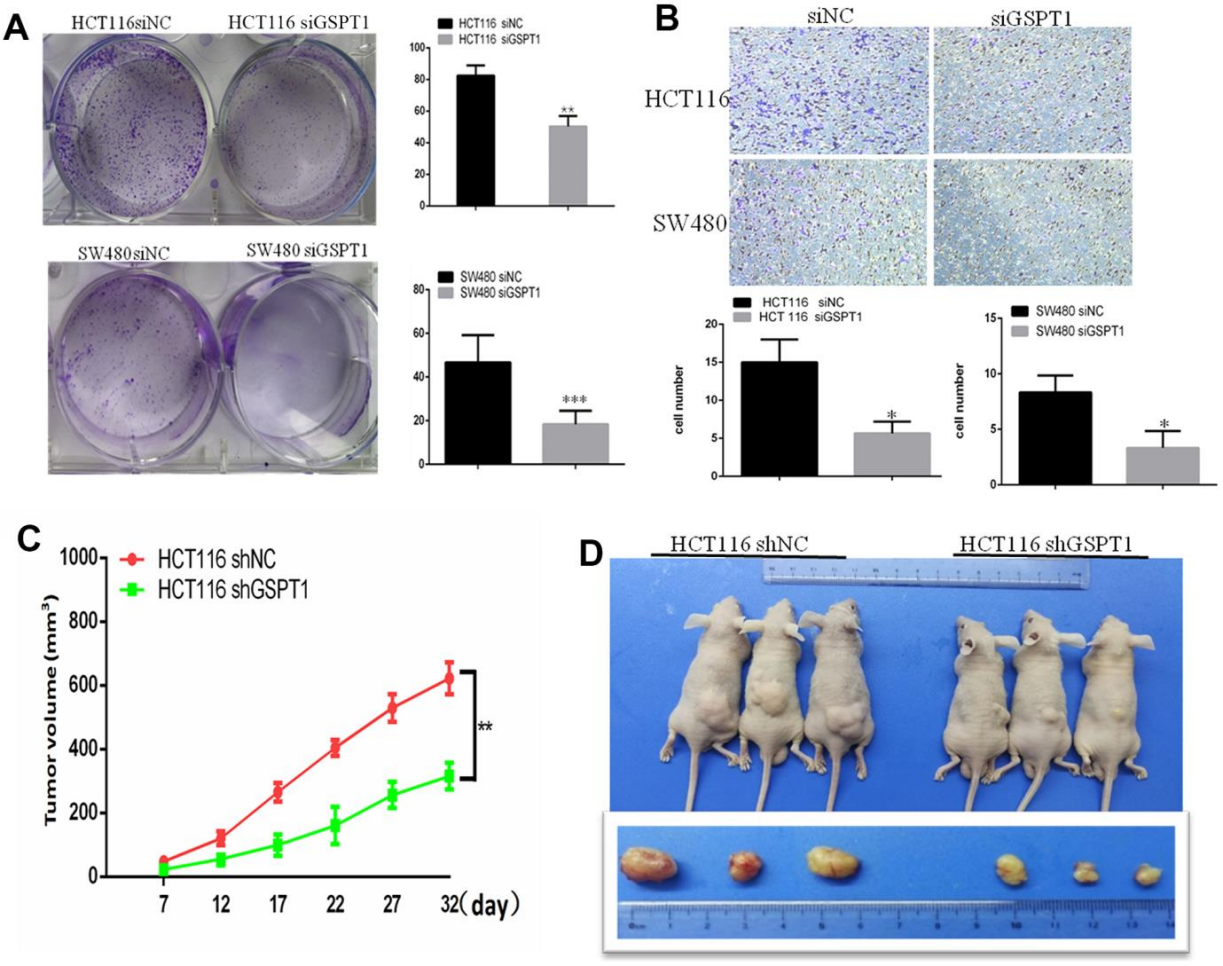
**GSPT1 knockdown inhibits colon cancer growth *in vitro* and *vivo*.** (**A**) Colony formation assay showed colonies numbers in the GSPT1 knockdown group were lower than those in NC group. (**B**) The cell invasion ability was detected by transwell assay. (**C**) Tumor volume of GSPT1 knockdown group was lower than NC group. (**D**) Tumor weight of the GSPT1 silence group was lower than NC group.

The effect of *GSPT1* interference on the colony formation ability of colon cancer cells was assessed using the plate clone formation test. The results showed that the number and size of HCT116 and SW480 colon cancer cell colonies were significantly reduced by siRNA interference compared with the control group (Figure 4A). Metastasis in colon cancer is characterized by increased cell motility, angiogenesis, and EMT. The role of *GSPT1* in advanced colon cancer was also investigated. As shown by the Transwell assay, *GSPT1* knockdown by si*GSPT1* dramatically reduced the invasion capacity of HCT116 and HT29 cells (Figure 4B). To detect the effect of *GSPT1* knockdown on colon cancer *in vivo*, tumor xenograft models were established in four-week-old BALB/c female nude mice inoculated with sh-*GSPT1* cells and control cells in the bilateral armpit. Compared to the control group, the tumors formed by GSPT1-knockdown colon cancer cells grew more slowly and were significantly smaller and lighter (Figure 4C, 4D). In addition, tumor tissues from the *GSPT1* knockdown group showed a lower degree of differentiation, abundant cytoplasm, no obvious boundary, large nuclear atypia, a high nucleocytoplasmic ratio, and an active mitotic phase relative to the control group (Supplementary Figure 1A). Immunohistochemistry confirmed that tumor tissues from the *GSPT1* knockdown group had fewer Ki67-positive cells than tissues from the control group (Supplementary Figure 1B).

### IP-MS reveal TRIM4 as potential downstream effectors of GSPT1

**Figure 5:**
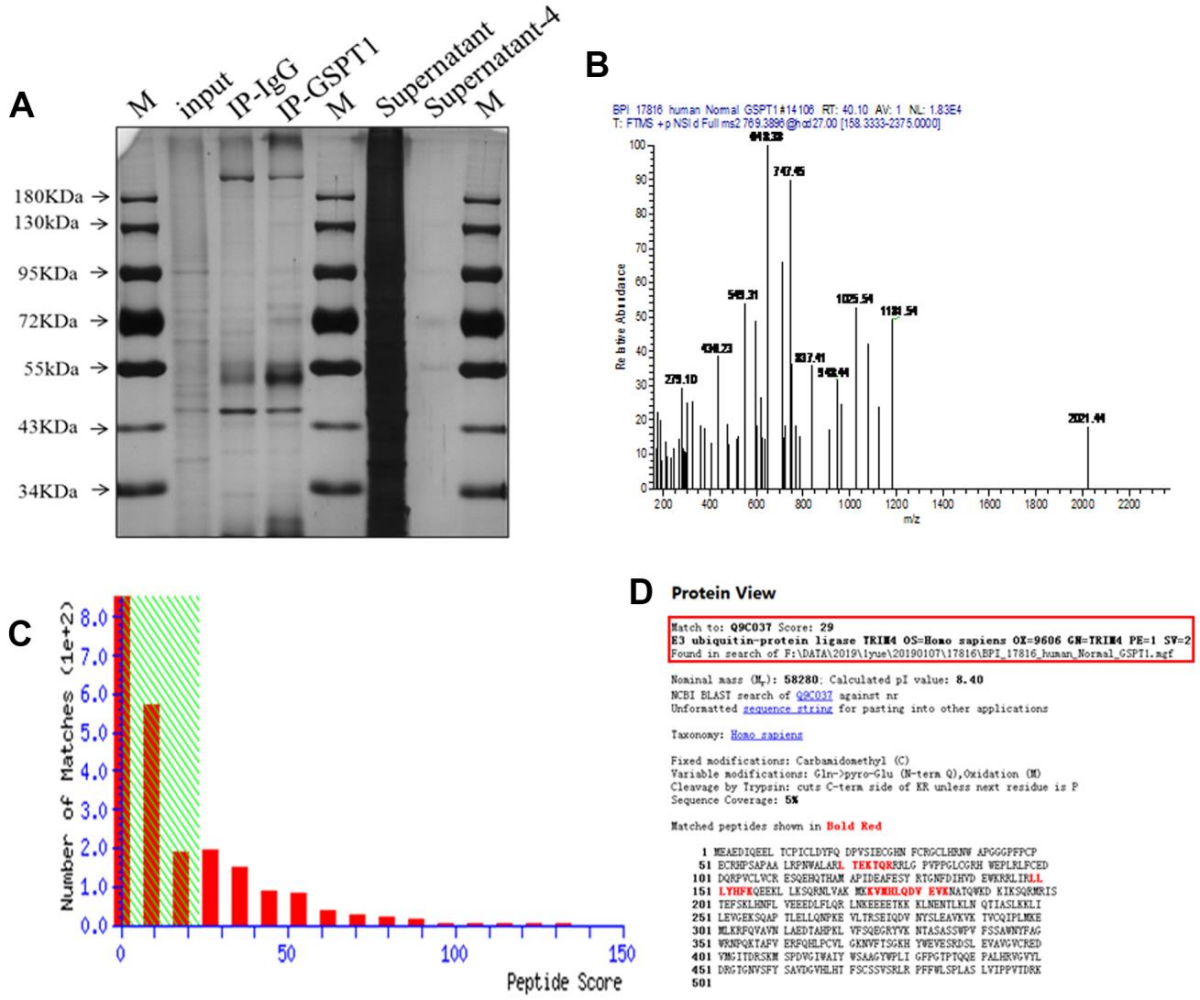
**IP-MS reveal TRIM4 as potential downstream effectors of GSPT1.** (**A**) The specific bands of IP-GSPT1 group and IP normal IgG group were detected by silver staining. (**B**) The peptide segments of specific bands were detected by mass spectrometry. (**C**) The fraction of peptide in the mass spectrometry results. (**D**) Analysis of the interaction between GSPT1 and TRIM4 in colon cancer.

To identify the binding protein of GSPT1, we carried out immunoprecipitation (IP) of GSPT1. The silver staining results showed that the IP-GSPT1 group had more specific bands than the IP normal IgG group, and so the two bands were detected by mass spectrometry (Figure 5A, 5B). The binding relationship between GSPT1 and E3 ubiquitin protein ligase (TRIM4) was determined by mass spectrometry (Figure 5C, 5D).

### GSPT1 drives colon cell tumorigenicity via the GSK3β axis

**Figure 6:**
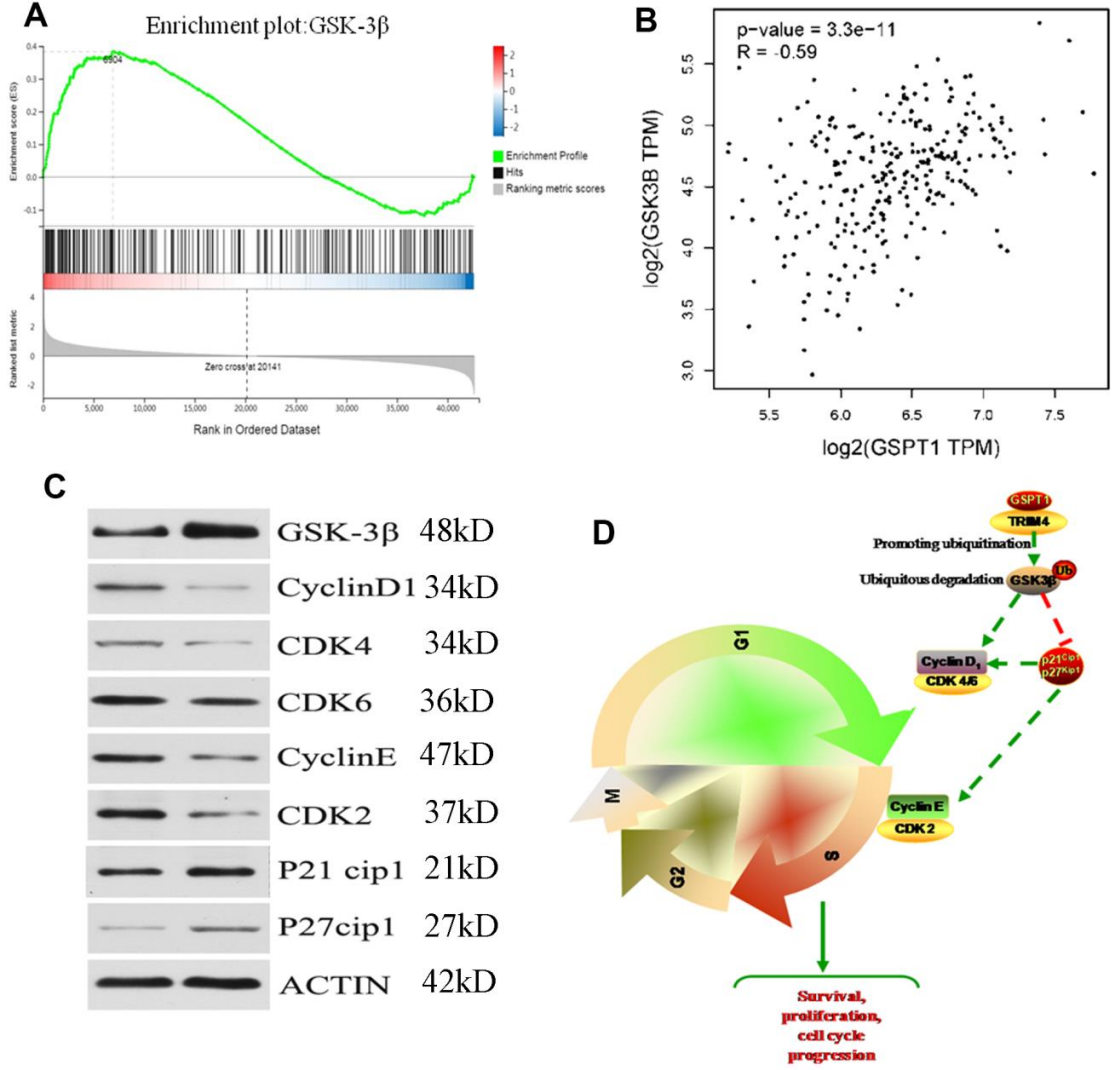
**GSPT1 drives the tumorigenicity of colon cells through the GSK3β axis.** (**A**) The gene set enrichment analysis of GSPT1. (**B**) Correlation analysis of GSPT1 and GSK in TCGA database. (**C**) GSPT1 negatively regulates GSK-3β signaling pathway. (**D**) The mechanism of GSPT1 in promoting the occurrence and development of colon cancer.

The results of Gene Set Enrichment Analysis (GSEA) suggested that the GSK-3β signaling pathway is the downstream signaling pathway of GSPT1 (Figure 6A). The expression of GSPT1 and GSK3β were negatively correlated in the TCGA database (Figure 6B). In order to investigate the relationship between GSPT1 and GSK-3β in colon cancer cells, the expression of GSK-3β was up-regulated after GSPT1 was silenced in HCT116 cells (Figure 6C). According to the relevant literature on the cell cycle pathway, GSK-3β is an important protein in the transition from G1 phase to S phase and its regulation of the cell cycle is realized by CyclinD1, CDK4/6, cyclinE, CDK2, etc. [14, 15]. After GSPT1 was silenced in HCT116 colon cancer cells, the expression of CyclinD1, CDK4/6, cyclinE, CDK2, p21, and p27 was detected by Western blot. The results showed that GSK-3β, p21, and p27 were up-regulated, whereas the expression of CyclinD1, CDK4/6, cyclinE, and CDK2 decreased (Figure 6C). Taken together, these results suggest that GSPT1 regulates CyclinD1, CDK4, and Cdk6 through GSK-3 β, and indirectly affects the expression of cyclinE and CDK2 through p21 and p27, thus regulating the transformation of colon cancer cells from the G1 phase to S phase and promoting tumor progression (Figure 6D).

## DISCUSSION

The incidence rate of colon cancer is increasing every year and poses a serious threat to human health. At present, the main treatment for colon cancer is still surgical treatment, combined with chemotherapy, radiotherapy, and targeted drug therapy [16–18]. The overall five-year survival rate for colon cancer is about 50%. The five-year survival rate at early diagnosis is 90%, and the incidence of distant metastasis is less than 10% [19]. Specific tumor biomarkers are urgently needed to help enable early diagnosis of colorectal cancer, selection of "personalized" treatment strategies, and to predict prognosis [20–22]. In the present study, we analyzed the sequencing data of colon cancer in the TCGA database and found that GSPT1 was abnormally highly expressed in colon cancer tissue and related to tumor size. We also analyzed the biological function of GSPT1 in colon cancer and the related molecular biological mechanism.

First, we silenced the expression of *GSPT1* in colon cancer cell lines HCT116 and SW480 by RNA interference. We detected HCT116 and SW480 cell proliferation by the CCK-8 assay at 24, 48, 72, and 96 hours after transfection with *GSPT1* siRNA. The results showed that HCT116 and SW480 proliferation began to be inhibited after 24 hours of siRNA transfection, with the most obvious inhibition at 72 hours. This result confirmed that the proliferation ability of colon cancer cells is significantly inhibited by *GSPT1* silencing. The results of the CCK-8 and colony formation assay confirmed that *GSPT1* promoted the growth of colon cancer cells. The results of the cell cycle and EdU assays showed that, after *GSPT1* was silenced, DNA synthesis of HCT116 and SW480 was inhibited and the cell cycle was significantly changed such that the proportion of G1 phase cells increased the proportion of S phase cells decreased. In addition, in HCT116 colon cancer cells transfected with the *GSPT1* overexpression plasmid, the proportion of G1 phase cells decreased, the proportion of S phase cells increased, and the ratio of G2/M phase cells increased. Thus, *GSPT1* was shown to play an important role in the transition from the G1 to S phase of the cell cycle. In HCT116 and SW480 cells, the apoptosis rate of the *GSPT1* silencing group was significantly higher than that of the negative control group, indicating that silencing *GSPT1* promoted cell apoptosis. By contrast, overexpression of *GSPT1* in HCT116 cells resulted in a significantly lower apoptosis rate relative to the control group, indicating that *GSPT1* inhibited the apoptosis. The results of the transwell Matrigel experiment showed that the number of cells passing through the transwell basement membrane decreased significantly after *GSPT1* was silenced by HCT116 and SW480, indicating that the invasion ability of colon cancer cells interfered with GSPT1 was inhibited. The *in vivo* tumorigenesis experiment with nude mice confirmed that the proliferation and tumorigenicity of colon cancer cells were significantly inhibited after *GSPT1* silencing. Notably, it has been reported that *GSPT1* promotes the proliferation, invasion, and migration of NSCLC cells, enhances tumorigenicity, and promotes the progression of lung cancer [23, 24]. In gastric cancer tissues, *GSPT1* is highly expressed, miRNA-144 expression is down-regulated, and GSPT1 expression is significantly increased. By inhibiting miRNA-144, *GSPT1* over-expression can promote the proliferation, invasion, and migration of gastric cancer cells, thereby promoting gastric cancer progression, which is consistent with the role of *GSPT1* in tumors [25]. It has also been reported that the overexpression of *GSPT1* is related to the specific expression of GGCn alleles in various cancer cells, and that it is a potential oncogene [26].

Immunoprecipitation (IP) technology is a classical method for studying protein-protein interactions based on the specific interaction between antibody and antigen; it is an effective method to determine the physiological interaction of two proteins in intact cells. Here, we identified the binding relationship between GSPT1 protein and E3 ubiquitin protein ligase (TRIM4) in colon cancer cells. E3 ubiquitination protein ligase TRIM4 is an important enzyme involved in ubiquitination. Protein ubiquitination is a common post-translational modification. The main function of ubiquitination is to participate in the degradation of substrate proteins and the elimination of abnormal proteins. Ubiquitination is involved in regulation of the cell cycle, proliferation, apoptosis, differentiation, metastasis, gene expression, transcription regulation, signal transmission, damage repair, inflammatory immunity, and other life activities. Ubiquitination is closely related to tumors. Using the IP assay, we found that GSPT1 and E3 ubiquitin ligase TRIM4 were bound. We also found that the expression levels of GSPT1 and GSK-3β were significantly negatively correlated. Thus, we speculate that GSPT1 and trim4 bind to ubiquitinate GSK-3β and play a related biological role.

In the present study, we found that *GSPT1* negatively regulated the GSK-3β signaling pathway in colon cancer cells. After *GSPT1* was silenced, the expression of GSK-3β, p21, and p27 increased, and the expression of CyclinD1, CDK4/6, cyclinE, and CDK2 decreased; these changes affected the progression of colon cancer cells from G1 phase to S phase and blocked the cell cycle. These results suggest that *GSPT1* and TRIM4 bind to ubiquitination to degrade GSK-3β, which may affect the cell cycle by affecting the GSK-3β signaling pathway. Ultimately, this promotes colon cancer cell proliferation, thus promoting the development of colon cancer. These findings suggest that GSPT1/TRIM4 ubiquitinated GSK-3β may be a potential molecular target for research, drug development, and clinical treatment of colon cancer.

## MATERIALS AND METHODS

### RNA sequencing data analysis

The level 3 data of RNA-seq and the corresponding clinical data of colon adenocarcinoma (COAD) were downloaded from the TCGA database (https://tcga-data.nci.nih.gov/tcga/). The raw gene counts data were extracted. Next, the R package DESeq was used to obtain normalized counts. Finally, genes with low expression levels (counts were 0 in more than 20% of samples) were eliminated. Using the R package glmperm combined with univariate Cox proportional hazards regression models, the relationship between GSPT1 expression and colon cancer stage was analyzed, and 10000 permutation tests were conducted to set the significant correlation threshold (*P* < 0.01).

### Colon cancer tissue samples and immunohistochemical staining

In total, 108 samples of colon cancer tissues and 12 samples of healthy colon tissues and adjacent normal tissues were purchased from Alenabio (Shanxi, China). The institutional approval number for this human study is YB-M-05-02. Tissue sections were deparaffinized, rehydrated with alcohol, and autoclaved at 121° C for 10 min in Target Retrieval solution, pH 6.0 (S2369; Dako, Glostrup, Denmark) for antigen retrieval. Hydrogen peroxide (3%) was then applied to these sections for 5 min at room temperature to block endogenous peroxidase. After washing twice with Tris buffer, the sections were incubated with GSPT1 antibodies (biorbyt; orb11324; US) (1:200 dilutions) for 1h at room temperature. The sections were washed twice with Tris buffer again and subsequently incubated with secondary horseradish peroxidase-conjugated antibodies (K5007; Dako; Denmark) for 30 min at room temperature, followed by incubation in 3,3-diaminobenzidine (K5007; Dako; Denmark) for 5 min, counterstained with Mayer’s hematoxylin for 90 sec, and mounted with Malinol.

Based on the immunohistochemical staining results, samples were classified as low- or high-level expression. The scoring system used to represent the percentage of positively stained tumor cells was as follows: 0, no positive tumor cells; 1, <10% positive cells; 2, 10–50% positive cells; and 3, >50% positive cells. The staining intensity was assigned as: 0, no staining; 1, weak staining; 2, moderate staining; and 3, strong staining. The staining index (SI) was calculated by multiplying the score obtained from the percentage of positive tumor cells and that obtained from the staining intensity in each sample. Under these conditions, seven possible SIs (0, 1, 2, 3, 4, 6, and 9) were generated for each sample. An SI >4 was considered high GSPT1 expression, while SI<3 was considered low expression.

### Cell culture and transfection

The human colon cancer cell lines HCT116 and SW620 were obtained from the cell bank of the Chinese Academy of Sciences. All cells were maintained in Dulbeco’s Modified Eagle Medium (DMEM, Gibco, Grand Island, NY, US) supplemented with 10% heat-inactivated fetal bovine serum (Gibco, Grand Island, NY, US) and 1% penicillin-streptomycin solution (Gibco, Grand Island, NY, US) in a humidified atmosphere of 5% CO_2_ at 37° C.

Specific small interfering RNAs (siRNAs) targeting GSPT1 (si-GSPT1) and negative control siRNA (si-NC) were acquired from GenePharma Co., Ltd. (Shanghai, China). Short hairpin RNA (shRNA) targeting GSPT1 and their sample control were designed by GenePharma (Shanghai, China). Oligos for expressing siRNA were then synthesized and inserted into the pGPH1 vector (GenePharma, Shanghai, China). For the RNA interference or GSPT1overexpression experiments, pGPH1 or pcDNA3.1-GSPT1 plasmids (1μg/well) and Lipofectamine 3000 (3μL/well) were mixed and applied to cells. For the siRNA experiments, 25pmol siRNA and sample control (NC) were mixed with 7.5μL Lipofectamine RNAiMAX reagent and applied for transfection. All transfected cells were cultured for 48h before the various assays were performed, unless indicated otherwise.

### RNA isolation and quantitative polymerase chain reaction (qRT-PCR)

Total RNA was extracted using TRIzol reagent (Takara, Dalian, China) according to the manufacturer’s instructions. Complementary DNA (cDNA) was synthesized from 1μg total RNA using the PrimeSipt RT reagent kit (Takara, Dalian, China). RT-PCR was performed using a Mastercycler® Nexus X2 (Eppendorf, Hamburg, Germany) with 95° C for 15 sec, 60° C for 60 sec, and 72° C for 40 sec (35 cycles). Glyceraldehyde-3-phosphate dehydrogenase (GAPDH) was used for the normalization of mRNA expression. All results were calculated using the 2^−ΔΔCt^ method.

### Cell counting kit (CCK)-8 assay

A total of 2 × 10^3^ transfected cells suspended in 100 μL culture medium were inoculated into 96-well plates. Cell proliferation was determined at 0, 24, 48, and 72 h after inoculation by adding 10μL CCK-8 solution (Sigma-Aldrich, St. Louis, MO, USA) into each well. Next, the plates were incubated at 37° C with 5% CO2 for an additional 2h. Absorbance at a wavelength of 450 nm was measured using a microplate reader (Multiscan MK3; Thermo Fisher Scientific, Inc.).

### Cell migration and invasion assay

Matrigel-coated (for the invasion assay) or non-coated (for the migration assay) 24-well Transwell culture inserts with a 5μm pore size were applied (CORNING, Corning, NY, USA). HCT116 or HT29 cells were transfected as described above and re-seeded onto the top of chamber of inserts with 400μL serum-free culture medium. For the bottom compartments, DMEM containing 20% FBS was used as a chemoattractant. After 24h, the inserts were collected, the non-invading cells on the upper surface were removed with a cotton swab, and the invaded cells on the lower surface were fixed with 4% paraformaldehyde and stained with crystal violet following the standard protocol.

### Cell cycle analysis

Compound-treated cells were collected, fixed in 70% pre-chilled ethanol at 4° C overnight, stained with propidium iodide (PI), and then examined by fluorescence-activated cell sorting (FACS) analysis (Becton-Dickinson, Mountain View, CA, USA).

### Mouse xenograft tumor models

Six BALB/c nude mice (4 weeks old) were purchased from the Beijing Experimental Animal Centre, China (approval number for lab animal studies: 20130312AX). HCT116 cells stably transfected with sh-GSPT1 (sh-GSPT1 group) or sh-NC (sh-NC group) were collected and subcutaneously injected into three BALB/c nude mice, respectively. Tumor growth was measured every three days using vernier calipers. Tumor volume was calculated as the product of 1/2×length×width×height. The mice were weighed and carefully monitored for the appearance of any side effects. At the end of the experiments, the mice were sacrificed for histological analysis of the lesions.

### Gene set enrichment analysis

Gene set enrichment analysis (GSEA) first generated an ordered list of all genes according to their correlation with *GSPT1* expression. GSEA was then carried out to elucidate the significant survival differences observed between the low and high *GSPT1* groups. The cutoff value of *GSPT1* expression was determined by the median value. Gene set permutations were performed 1000 times for each analysis. The expression level of *GSPT1* was used as a phenotype label. The leading edge subset was the most important member of the gene. If the enrichment score is positive, it is the gene on the left side of the peak; if the enrichment score is negative, it is the gene on the right side of the peak.

### Statistical analysis

Statistical analyses were performed using SPSS 20.0 (IBM, USA). Kaplan–Meier curves were generated by GraphPad Prism 7. Data are reported as mean±SD. T-tests were used to assess differences between two groups of independent samples. The relationship between GSPT1 expression and clinical characteristics was analyzed by the χ^2^ test. The correlation between gspt1 and GSK was measured by the Pearson correlation coefficient: an r value greater than 0 indicates positive correlation, while r value less than 0 indicates negative correlation. The Kaplan–Meier method and log-rank test were used for the survival rate analysis. For all analyses, a *p* value < 0.05 was considered statistically significant.

### Availability of data and materials

The datasets used and/or analyzed in the current study are available from the corresponding author on reasonable request.

### Ethics approval and consent to participate

The study was approved by the ethical committee of Mianyang Central Hospital.

## Supplementary Material

Supplementary Figure 1
