# Supplementary material for: Identification of GSPT1 as prognostic biomarker and promoter of malignant colon cancer cell phenotypes via the GSK-3β/CyclinD1 pathway
Source: Aging (Albany NY). 2021 Apr 4;13(7):10354–68. doi: 10.18632/aging.202796 (PMC8064227; doi:10.18632/aging.202796)
Supplement: Supplementary Figure 1 [file aging-13-202796-s001.pdf]

SUPPLEMENTARY FIGURE

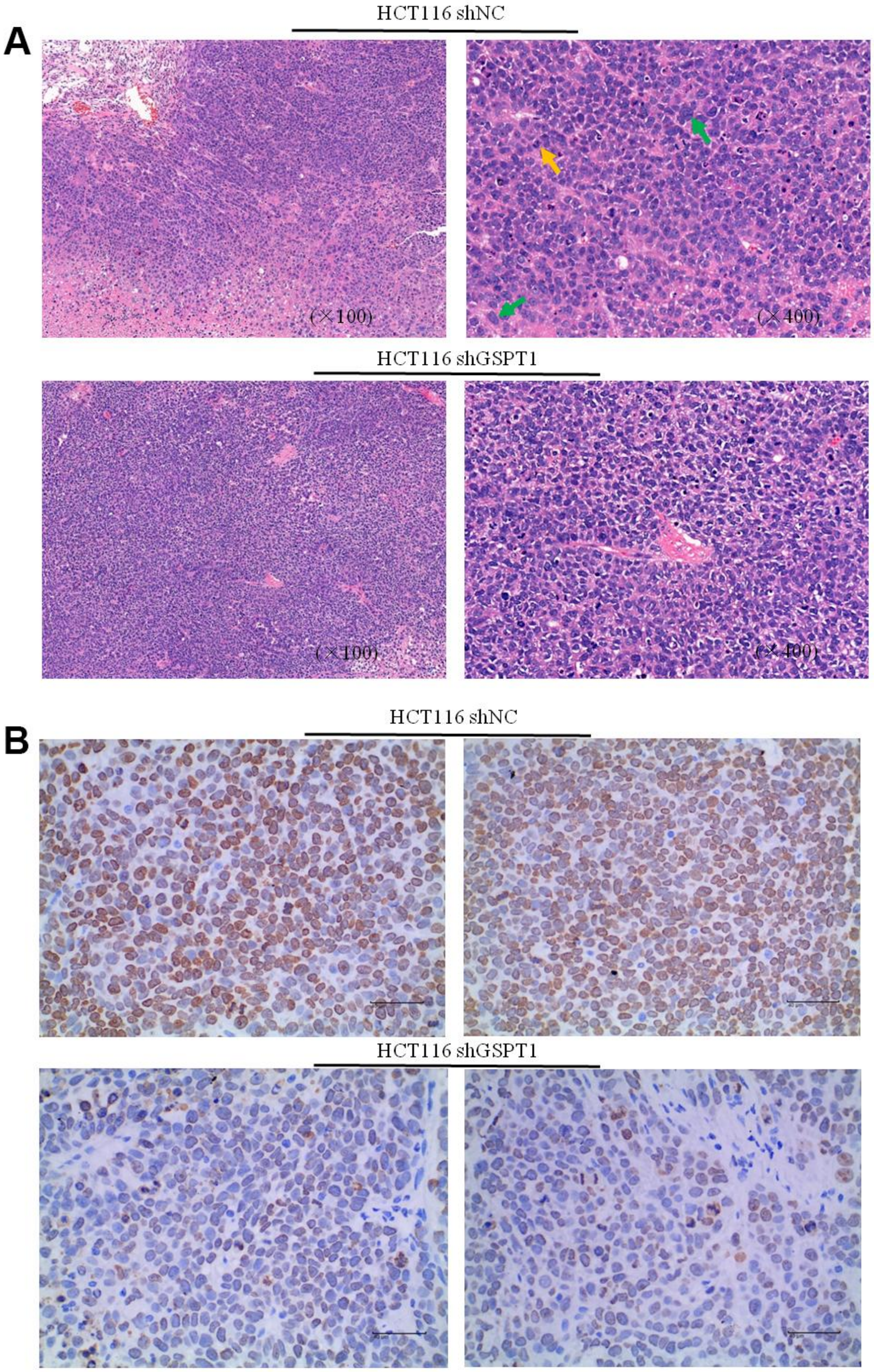

Supplementary Figure 1. Silencing of GSPT1 inhibits HCT-116 cell growth in vivo. (**A**) HE staining of tumor samples from knockdown GSPT1 groups and control HCT116 groups. The nuclear atypia of tumor cells was large(↑), Mitotic phase(↑). (**B**) Ki67 immunostaining of tumor samples from knockdown GSPT1 groups and control HCT116 groups.
